# Using the minimum description length principle to reduce the rate of false positives of best-fit algorithms

**DOI:** 10.1186/s13637-014-0013-2

**Published:** 2014-07-03

**Authors:** Jie Fang, Hongjia Ouyang, Liangzhong Shen, Edward R Dougherty, Wenbin Liu

**Affiliations:** 1 Department of Physics and Electronic information engineering, Wenzhou University, Wenzhou, 325035 Zhejiang China; 2 Department of Electrical and Computer Engineering, Texas A&M University, College Station, 33101 TX USA; 3 Center for Bioinformatics and Genomics Systems, College Station, 33101 TX USA

**Keywords:** Boolean network, Best-fit, Minimum description length principle, Conditional mutual information

## Abstract

The inference of gene regulatory networks is a core problem in systems biology. Many inference algorithms have been proposed and all suffer from false positives. In this paper, we use the minimum description length (MDL) principle to reduce the rate of false positives for best-fit algorithms. The performance of these algorithms is evaluated *via* two metrics: the normalized-edge Hamming distance and the steady-state distribution distance. Results for synthetic networks and a well-studied budding-yeast cell cycle network show that MDL-based filtering is more effective than filtering based on conditional mutual information (CMI). In addition, MDL-based filtering provides better inference than the MDL algorithm itself.

**Electronic supplementary material:**

The online version of this article (doi:10.1186/s13637-014-0013-2) contains supplementary material, which is available to authorized users.

## 1 Introduction

A key goal in systems biology is to characterize the molecular mechanisms that govern specific cellular behavior and processes. Models of gene regulatory networks run the gamut from coarse-grained discrete networks to detailed descriptions of such networks by stochastic differential equations [1]. Boolean networks and the more general class of probabilistic Boolean networks are among the most popular approaches for modeling gene networks because they provide a structured way to study biological phenomena (e.g., the cell cycle) and diseases (e.g., cancer), ultimately leading to systems-based therapeutic strategies. The inference of gene networks from high-throughput genomic data is an ill-posed problem known as reverse engineering. It is particularly challenging when dealing with small sample sizes because the number of variables in the system (e.g., the number of genes) typically is much greater than the number of observations [2]. Many inference algorithms have been proposed to elucidate the regulatory relationships between genes, such as Reveal [3], ARACNE [4], the minimum description length principle (MDL) [5]–[9], the coefficient of determination (CoD) [10],[11], and the best-fit extension [12],[13].

False positives are a common problem in inference, especially when dealing with small sample sizes and noisy conditions. In fact, false positives are a kind of structural redundancy. Given three genes, *x*_1_, *x*_2_, and *x*_3_, they may interact in a chain-like manner, such as *x*_1_ → *x*_2_ → *x*_3_ or *x*_1_ ← *x*_2_ ← *x*_3_; or in a hub-based way, such as *x*_1_ → *x*_2_ ← *x*_3_ or *x*_1_ ← *x*_2_ → *x*_3_. Indirect interactions between two genes may produce some correlation in their expression data, which can lead to a false regulation detection by inference algorithms. The data-processing inequality (DPI) was first used in ARACNE, which aims to reduce the false positives produced by chain interaction [4]. Later, conditional mutual information (CMI) was proposed to tackle the false positives produced by both the chain-like and hub-based interactions [14]. Because the conditioning gene, *x*_2_, is usually not known, a greedy search strategy was adopted to check if the CMI between *x*_1_ and *x*_3_ conditioned on some other genes was below a given threshold. To check the CMI on other unrelated genes is problematic. Not only is it computationally burdensome, it also suffers from an enormous multiple-comparisons problem. Moreover, since the interaction strength between genes generally varies a lot, their being both strong and weak interactions, how to set an appropriate threshold is a key problem.

A recent study shows that the best-fit algorithm appears to give the best results for recovering regulatory relationships in comparison to the aforementioned algorithms [15]. In the present paper, we propose to reduce the false positives of the best-fit algorithm by using the MDL principle. Simulation results show that it is more effective than the CMI-based method and can reduce the false positives in the MDL algorithm in [5]. In effect, the false-positive reducing procedure acts as a filter for removing false positives.

The aim of filtering in the present framework is to reduce the number of false positive connections. As with any false-positive reducing algorithm, this will invariably increase the number of false negatives, meaning more missing connections. Thus, two questions must be addressed. First, what benefits accrue from reducing the number of false positives? Second, does the increase in false negatives significantly impact inference performance?

A salient problem in translational genomics is the utilization of gene regulatory networks in determining therapeutic intervention strategies [2],[16],[17]. A big obstacle in deriving optimal treatment strategies from networks is the computational complexity arising directly from network complexity. Hence, significant effort has been focused on network reduction [18],[19]. As with any compression scheme, reduction methods sacrifice information in return for computational tractability. Because genes are removed from the network based upon their regulatory relations with other genes, false positives are particularly troublesome. First, they increase the amount of reduction necessary and second, they compete with true positive connections for retention in the reduced network. While it is true that an increase in false negatives is not beneficial, a missing connection creates no additional computational burden (in fact, reduces computation) and plays no role in the reduction procedure.

Now, for the caveat, all of this is fine, so long as the accuracy of the original inference algorithm is not adversely impacted. Practically, this means that, relative to some distance function between a ground-truth network and an inferred network (which quantifies inference accuracy), the distance is not increased when using the modified false-positive reducing algorithm in place of the original algorithm. In this paper, we will consider two distance functions, one based on the hamming distance between the ground-truth and inferred networks and the other based on the difference between the steady-state distributions of the ground-truth and inferred networks.

This paper is organized as follows: Background information and necessary definitions are given in Section 2. The implementation of MDL, the best-fit algorithm, and CMI- and MDL-based filtering is then introduced in Section 3. Results from simulated networks and from the cell cycle model of budding yeast are presented in Section 4. Finally, concluding remarks are given in Section 5.

## 2 Background

### 2.1 Boolean networks

A Boolean network *G*(*V*, *F*) is defined by a set of nodes *V* = {*x*_1_, …, *x*_*n*_}, *x*_*i*_ ∈ {0, 1}, and a set of Boolean functions *F* = {*f*_1_, …, *f*_*n*_}, $f_{i}:{\left\{0,1\right\}}^{k_{i}}\to \left\{0,1\right\}$ Each node *x*_*i*_ represents the expression state of a gene, where *x*_*i*_ = 0 means that the gene is off and *x*_*i*_ = 1 means it is on. To update its value, each node *x*_*i*_ is assigned a Boolean function $f_{i}\left(x_{i1},\ldots ,x_{ik_{i}}\right)$with *k*_*i*_ specific input nodes. Under the synchronous updating scheme, all genes are updated simultaneously according to their corresponding update functions. The network's state at time *t* is represented by a binary vector *x*(*t*) = (*x*_1_(*t*), …, *x*_*n*_(*t*)). In the absence of noise, the state of the system at the next time step is

1 $$x\left(t+1\right)=F\left(x_{1}\left(t\right),\ldots ,x_{n}\left(t\right)\right).$$

The long-term behavior of a deterministic Boolean network depends on the initial state. The network will eventually settle down and cycle endlessly through a set of states called an *attractor cycle*. The set of all initial states that reach a particular attractor cycle forms the *basin of attraction* for the cycle. Following a random perturbation, the network may escape an attractor cycle, be reinitialized, and then begin its transition process anew. For a Boolean network with perturbation, its corresponding Markov chain possesses a steady-state distribution. It has been hypothesized that attractors or steady-state distributions in Boolean formalisms correspond to different cell types of an organism or to cell fates. In other words, the phenotypic traits are encoded in the attractors or steady-state distribution [1].

### 2.2 Best-fit extension

One approach to infer Boolean networks is to search a consistent rule from examples, the so-called consistency problem [20]. Owing to noise in gene-expression profiles, we relax it to the called best-fit extension problem, which has been extensively studied for many function classes [21]. We briefly introduce the best-fit extension problem for Boolean functions. A partially defined Boolean function (pdBf) is defined by two sets, T, F ⊆ {0, 1}^*n*^, where T and F represent the set of true and false vectors, respectively. A function *f* is called an *extension* of pdBf(T, F) if T ⊆ T(*f*) = {*x* ∈ {0, 1}^*n*^ : *f*(*x*) = 1} and F ⊆ F(*f*) = {*x* ∈ {0, 1}^*n*^ : *f*(*x*) = 0}. The magnitude of the error of function *f* is

2 $$\varepsilon \left(f\right)=T\cap F\left(f\right)+F\cup T\left(f\right).$$

The best-fit extension aims to find two subsets T* and F* such that T* ∩ F* = *ϕ* and T* ∪ F* = T ∪ F, for which the function pdBf(T*, F*) has an extension in some class *C* of Boolean functions such that T* ∩ F + F * ∪ T is minimized. Clearly, any extension *f* ∈ *C* of pdBf (T*, F*) has minimum error magnitude [12],[13].

### 2.3 Conditional mutual information

Mutual information (MI) is a general measurement that can detect nonlinear dependence between two random variables *X* and *Y*. For discrete-valued random variables, the one-time-lag MI from *X*_*t*_ to *Y*_*t* + 1_ is given by

3 $$I\left(Y_{t+1};X_{t}\right)=H\left(Y_{t+1}\right)-H\left(Y_{t+1}|X_{t}\right)$$

where *H*(•) denotes entropy and *X*_*t*_ and *Y*_*t* + 1_ are two equal-length vectors. The conditional mutual information (CMI) from *X*_*t*_ to *Y*_*t* + 1_ given *Z*_*t*_ is

4 $$I\left(Y_{t+1};X_{t}|Z_{t}\right)=H\left(Y_{t+1}|Z_{t}\right)-H\left(Y_{t+1}|X_{t},Z_{t}\right),$$

and quantifies the reduction in the uncertainty of *Y*_*t*+1_ due to knowledge of *X*_*t*_ given *Z*_*t*_. In the chain-like or hub-based scenarios, genes *X*_*t*_ and *Y*_*t*+1_ should be independent given the intermediate or hub gene *Z*_*t*_, which means that *I*(*X*_*t*_; *Y*_*t* + 1_|*Z*_*t*_) = 0.

### 2.4 Minimum description length principle

A fundamental principle in model selection is the minimum description length (MDL) principle, which states that we should choose the model that gives the shortest description of the data. The ‘two-part MDL’ developed by Rissanen consists of writing the description length of a given model applied to a data set as the sum of the code length for describing the model and the code length for describing the data set fit by the model [22]

5 $$L=L_{M}+L_{D}.$$

There are various ways to encode the model-coding length *L*_*M*_ and the data-coding length *L*_*D*_. Given a time series of length *m*, Zhao et al. proposed to encode *L*_*M*_ and *L*_*D*_ as [5]

6 $$L_{M}=\tau \sum_{i=1}^{n}\left\{d_{i}*k_{i}+d_{f}*2^{k_{i}}\right\},$$

7 $$L_{D}=-\sum_{i=1}^{n}\sum_{t=1}^{m-1}\log p\left(x_{i}\left(t+1\right)|x_{i1}\left(t\right)\cdots x_{ik_{i}}\left(t\right)\right),$$

where *τ* is a free parameter to balance the model- and data-coding lengths, *n* and *m* are the number of genes and time points. *d*_*i*_ = ⌈ log_2_*n*⌉ and *d*_*f*_ = ⌈ log_2_*m*⌉ denote the number of bits needed to code an integer and a floating-point number, respectively.

## 3 Implementation

Based on the common assumption that genetic regulatory networks are sparsely connected, we restrict simulated Boolean networks to a scale-free topology with maximal connectivity *K* = 4 and average connectivity *k* = 2. The best-fit algorithm searches for the best-fit function for each gene by exhaustively searching for all combinations of potential regulator sets. The search space grows exponentially with the number of genes. In practice, the limit *k*_*i*_ ≤ 3 is generally applied to mitigate model complexity. In this paper, we restrict best-fit-algorithm searches to combinations of 1, 2, or 3 possible regulators. The combinatorial set with the smallest error is then selected as the regulatory set. We call this best-fit-I. In practice, the minimal error predictor set may not unique. We employ the heuristic that each of them can be viewed as fitting the target gene in a different way and if one gene occurs frequently in those sets, then it is highly likely to be a true regulatory gene. Thus, we can determine the regulatory set by applying the majority rule in these sets. Here, we refer to this algorithm as best-fit-II.

Then CMI and MDL criteria are used to filter false-positive connections. For each regulatory connection, if the CMI for one of the remaining genes is less than 0.005, then the gene is deleted; otherwise, it remains. The MDL criterion is applied to each target gene *x*_*i*_. Given its parent set, *Pa*(*x*_*i*_), we delete the regulatory gene *x*_*j*_ ∈ *Pa*(*x*_*i*_) that can maximally reduce its coding length *L*_*i*_ for each point in time, repeating this process until the deletion of one regulatory gene causes *L*_*i*_ to increase. We implement an MDL inference algorithm by directly searching the combination of 1, 2, or 3 possible regulators with minimal coding length *L*_*i*_. The free parameter *τ* in Equation 6 is set to 0.2.

We have analyzed CMI- and MDL-based filtering by using both synthetic networks as well as the well-studied cell-cycle model known as the budding-yeast network. We compare them with the ground-truth network according to the following two distances [15],[23]:

1. The normalized-edge Hamming distance:

8 $$\mu_{\mathrm{ham}}^{e}=\frac{\mathrm{FN}+\mathrm{FP}}{P},$$

where *FN* and *FP* represent the number of false-negative and false-positive wires, respectively, and *P* represents the total number of positive wires. This Hamming distance reflects the accuracy of the recovered regulatory relationships.

(2) The steady-state distribution distance:

9 $$\mu^{\mathrm{ssd}}=\sum_{k=1}^{2n}\left|\pi_{k}-\pi_{k}^{\prime }\right|,$$

where *π*_*k*_ and $\pi_{k}^{\prime }$ are the steady-state probabilities state *x*_*k*_ in the ground-truth and inferred network, respectively. The steady-state distribution distance reflects the degree to which an inferred network approximates the long-run behavior of the ground-truth network.

## 4 Results and discussion

### 4.1 Simulation on synthetic networks

**Figure 1:**
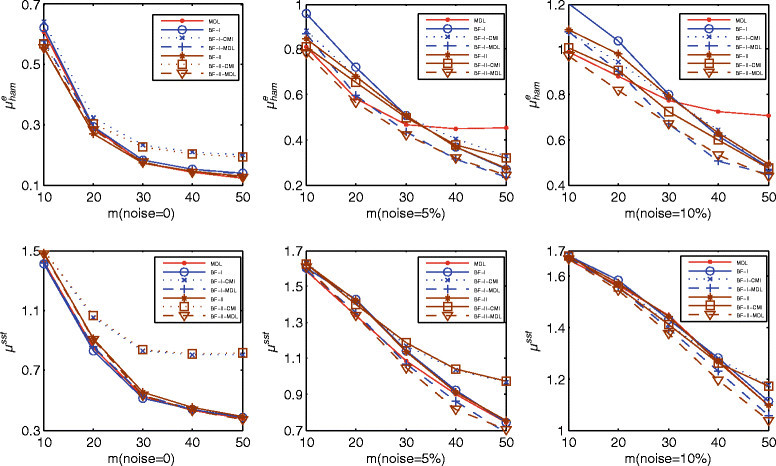
**Comparison of normalized-edge Hamming distance** $\mu_{\mathrm{ham}}^{e}$ **and steady-state distribution distance** **μ** ^**ssd**^ **with 0%, 5%, and 10% noise for MDL, best-fit-I, and best-fit-II filtered by CMI and MDL.**

We generated 1,000 random *n* = 10 genes and for each network generated a random sample of *m* = 10, 20, 30, 40, and 50 time points. As it is hard to obtain one time series with required length, we adopt the following sampling strategy: (1) select several start states which are the farthest from their attractor; (2) run each start state to its attactor; (3) select one path as a time series, if its length is shorter than required, add another path in it until we have required length of time points. We added 5% and 10% noise to these samples to investigate the effect of noise. The perturbation probability to calculate the steady-state distribution was set to *p* = 0.0001. In Table 1, we list the average number of true-positive and false-positive connections for various noise intensities. Figure 1 shows the average performance of the MDL, best-fit-I, and best-fit-II filtered by CMI and MDL for 0%, 5%, and 10% noise. As a whole, the performance of these algorithms increases as sample size increases from 10 to 50. This result is easy to understand: the more data we have, the better the inferred results.

**Table 1:** **Average number of true-positive and false-positive connections for MDL, best-fit-I, and best-fit-II filtered by CMI and MDL**

Noise (%)	Algorithm	***m***= 10		***m***= 20		***m***= 30		***m***= 40		***m***= 50	
		TP	FP	TP	FP	TP	FP	TP	FP	TP	FP
0	MDL	10.9	3.0	15.4	1.1	17.0	0.5	17.5	0.3	17.7	0.1
	BF-I	11.4	3.8	15.8	1.6	17.1	0.7	17.4	0.4	17.5	0.3
	BF-I-CMI	10.4	3.2	14.8	1.3	15.9	0.6	16.2	0.4	16.3	0.3
	BF-I-MDL	11.0	2.6	15.4	1.2	16.9	0.6	17.3	0.4	17.5	0.2
	BF-II	11.7	2.8	16.1	1.5	17.3	0.7	17.6	0.6	17.7	0.3
	BF-II-CMI	10.9	2.3	15.2	1.3	16.1	0.6	16.4	0.4	16.4	0.2
	BF-II-MDL	10.8	1.9	15.3	0.9	16.9	0.4	17.5	0.3	17.6	0.2
5	MDL	9.5	5.8	14.1	5.8	16.2	5.5	17.0	5.9	17.4	6.4
	BF-I	10.0	9.1	14.5	8.9	16.4	6.5	17.0	4.3	17.3	2.7
	BF-I-CMI	9.1	6.7	13.5	7.1	15.2	5.2	15.7	3.8	15.9	2.5
	BF-I-MDL	9.4	6.8	14.2	6.0	16.3	5.0	16.9	3.1	17.3	2.0
	BF-II	10.4	7.3	14.9	8.5	16.6	6.8	17.3	4.6	17.5	3.0
	BF-II-CMI	9.7	5.9	14.0	7.1	15.4	5.3	16.0	3.5	16.0	2.4
	BF-II-MDL	9.3	4.9	14.0	5.3	16.2	4.7	17.0	3.4	17.3	2.2
10	MDL	8.3	8.1	12.8	10.4	15.1	10.6	16.2	10.7	16.9	11.0
	BF-I	8.8	12.9	13.0	13.7	15.1	11.1	16.3	8.6	16.8	6.4
	BF-I-CMI	7.9	9.4	12.1	11.0	13.9	9.7	14.9	7.7	15.3	4.5
	BF-I-MDL	8.1	9.6	12.6	10.7	15.0	8.4	16.2	6.3	16.8	5.8
	BF-II	9.2	10.9	13.5	13.1	15.6	11.4	16.6	9.2	17.1	7.0
	BF-II-CMI	8.4	8.5	12.6	10.8	14.4	8.9	15.1	7.2	15.5	5.0
	BF-II-MDL	8.1	7.5	12.6	9.0	15.1	8.5	16.3	6.9	16.9	5.6

BF, best-fit.

Examination of the table reveals several trends. First, MDL-based filtering (dashed lines in Figure 1) always performs better than CMI-based filtering (dotted lines in Figure 1). MDL-based filtering aims to reduce the redundancy of a model according to the MDL principle, whereas CMI-based filtering attains reduction by blindly checking if the CMI of a connection conditioned on all other genes is below a given threshold. The results indicate that the former approach is superior to the latter. According to Table 1, on the whole, MDL-based filtering retains more true connections and deletes more false connections than CMI-based filtering.

Second, the performances of MDL, best-fit-I, and best-fit-II are very similar when used with noiseless data. In this case, the MDL algorithm gives a model with *L*_*D*_ = 0, which also corresponds to the zero-error model obtained by best-fit-I. In addition, MDL-based filtering results in little improvement over the best-fit algorithms. However, their performance is strongly related to sample size when the data are noisy. Specifically, for sample size less than 30, MDL performs better than best-fit-I and best-fit-II based on the average Hamming-edge distance $\mu_{\mathrm{ham}}^{e}$. But MDL performs worse than best-fit-I and best-fit-II for sample sizes lager than 30, because the structural regularization of MDL is beneficial only for small sample sizes whereas it leads to overfitting for large sample sizes. From Table 1, we see that, compared with best-fit-I and best-fit-II, the rate of false positives is relatively low for MDL with small sample sizes and relatively high for MDL with large sample sizes. Concerning the steady-state distribution distance *μ*^ssd^, MDL performs better than best-fit-I and best-fit-II for data with 5% noise, but the performance of these algorithms becomes equivalent for data with 10% noise. This result may be due to the noise not only deteriorating the inference of the regulatory relationships, but also deteriorating the interaction Boolean functions, which strongly influence *μ*^ssd^.

Third, for noisy situations, based on $\mu_{\mathrm{ham}}^{e}$ and μ^ssd^, not only does MDL-based filtering not degrade performance, it improves the performance of best-fit-I and best-fit-II, with the performance for best-fit-II being slightly better than that of best-fit-I. One reason for this result may be that best-fit-II infers more true-positive connections and less false-positive connections in small-sample situations (see Table 1). It is interesting that, in noisy situations, MDL-based filtering can even outperform the MDL algorithm across all sample sizes. In essence, the two methods are totally different because the former aims to reduce the structural redundancy of the minimal-error model obtained by the best-fit algorithm, whereas the latter aims to search the model with the minimum coding length *L*. From the point of view of the MDL principle, the coding length *L* of MDL-based filtering may not be the minimum length. Because MDL-based filtering combines both the best-fit algorithm and the MDL principle, it reduces structural redundancy and overcomes the over-fitting in large-sample-size situations.

### 4.2 Cell cycle model of budding yeast

**Figure 2:**
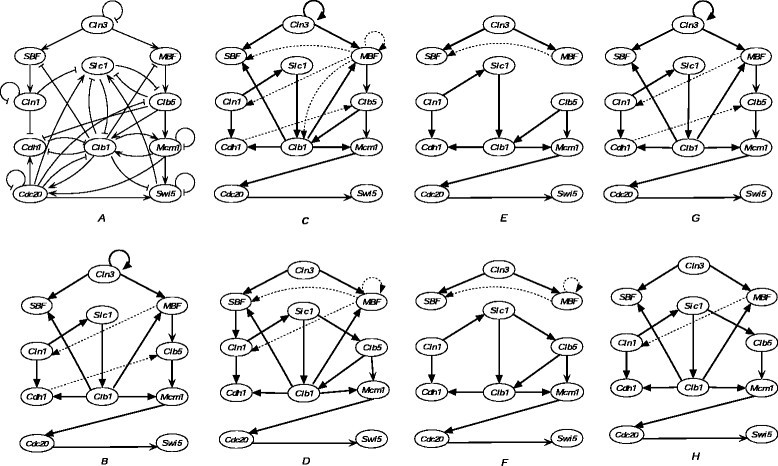
**Simplified cell-cycle network of budding yeast and the inferred networks from time-series in Table** 2**.** **(A)** Simplified cell-cycle network of budding yeast. Arrows are positive regulation, “T” lines are negative regulation, “T” loops are self-degradation. **(B)** Network inferred by MDL. **(C)** Network inferred by best-fit-I. **(D)** Network inferred by best-fit-II. **(E)** Network inferred by best-fit-I filtered by CMI. **(F)** Network inferred by best-fit-II filtered by CMI. **(G)** Network inferred by best-fit-I filtered by MDL. (H) Network inferred by best-fit-II filtered by MDL. From panel **(B)** to **(H)**, the bold solid lines are the correctly inferred regulatory relations, while the light dashed lines are the incorrectly inferred regulatory relations.

The cell cycle is a vital biological process in which one cell grows and divides into two daughter cells. It consists of four phases, G1, S, G2, and M, and is regulated by a highly complex network that is highly conserved among the eukaryotes. From the 800 genes involved in the cell cycle process of budding yeast, Li et al. constructed a network of 11 key regulators: Cln3, MBF, SBF, Cln1, Cdh1, Swi5, Cdc20, Clb5, Sic1, Clb1, and Mcm1 [24]. This Boolean network model, shown in Figure 2A, has an attractor whose biggest basin corresponds to the biological G1 stationary state. The temporal sequence in Table 2 is a pathway from this basin that follows the biological trajectory of the cell cycle network.

**Table 2:** **Temporal evolution of state for the cell cycle**

Time	Cln3	MBF	SBF	Cln1	Cdh1	Swi5	Cdc20	Clb5	Sic1	Clb1	Mcm1	Phase
1	1	0	0	0	1	0	0	0	1	0	0	Start
2	0	1	1	0	1	0	0	0	1	0	0	G1
3	0	1	1	1	0	0	0	0	1	0	0	G1
4	0	1	1	1	0	0	0	0	0	0	0	G1
5	0	1	1	1	0	0	0	1	0	0	0	S
6	0	1	1	1	0	0	1	1	0	1	1	G2
7	0	0	0	1	0	1	1	1	0	1	1	M
8	0	0	0	0	0	1	1	0	0	1	1	M
9	0	0	0	0	0	1	1	0	1	1	1	M
10	0	0	0	0	0	1	1	0	1	0	1	M
11	0	0	0	0	1	1	0	0	1	0	0	M
12	0	0	0	0	1	0	0	0	1	0	0	M
13	0	0	0	0	1	0	0	0	1	0	0	G1

We applied MDL, best-fit-I, and best-fit-II filtered by CMI and MDL to the artificial time-series data in Table 2. The inferred networks are shown in Figure 2. Figure 2B shows the network inferred by the MDL algorithm, which is the best network. Figure 2C,D has the same number of true-positive connections, with the latter having fewer false-positive connections. This result demonstrates that the method of selecting regulatory genes in best-fit-II is superior to using best-fit-I. Compared with Figure 2E,F, which was filtered by CMI from Figure 2C,D, Figure 2G,H filtered by MDL have more true connections, whereas the number of false-positive connections are about the same. Furthermore, we can see that the networks resulting from CMI-based filtering have two disconnected subgraphs, whereas the network resulting from MDL is a connected graph. This result shows that MDL-based filtering is more effective than CMI-based filtering. In fact, Figure 2G shows the same result as in Figure 2B, which is the best result.

We also ran 100 simulations with 5% and 10% noise for the pathway under consideration. Table 3 lists the average number of true positives and false positives, the normalized Hamming-edge distance $\mu_{\mathrm{ham}}^{e}$ and the steady-state distribution distance μ^ssd^. The results are consistent with those of the simulated networks (Figure 1) and they demonstrate that MDL-based filtering is effective for samples containing a small amount of noise.

**Table 3:** **Comparison of MDL, best-fit-I, and best-fit-II with CMI- and MDL-based filtering for yeast-pathway data**

Algorithm	Noise = 0				Noise = 5%				Noise = 10%			
	TP	FP	$\mu_{\mathrm{ham}}^{e}$	μ^ssd^	TP	FP	$\mu_{\mathrm{ham}}^{e}$	μ^ssd^	TP	FP	$\mu_{\mathrm{ham}}^{e}$	μ^ssd^
MDL	14	2	0.65	1.31	11.5	9	0.93	1.42	8.9	12.5	1.11	1.45
BF-I	15	5	0.71	1.25	12.2	11.9	0.99	1.44	9.8	18.4	1.25	1.49
BF-I-CMI	11	1	0.71	1.43	10.4	9	0.96	1.47	8.3	14	1.17	1.51
BF-I-MDL	14	2	0.65	1.17	10.8	8.5	0.93	1.43	8.6	13.1	1.13	1.48
BF-II	15	3	0.65	1.41	12.4	10.4	0.94	1.45	10.6	16.5	1.17	1.48
BF-II-CMI	12	2	0.71	1.46	11	8.7	0.93	1.47	8.3	12.4	1.12	1.50
BF-II-MDL	13	1	0.65	1.36	11.1	7.7	0.9	1.42	9.2	11.9	1.08	1.44

## 5 Conclusion

Reducing the rate of false positives is an important issue in network inference. In this paper, we address this question by using the minimum description length (MDL) principle. Specifically, we apply the MDL measurement technique proposed by Zhao et al. to filter the model obtained by two best-fit algorithms (best-fit-I and best-fit-II). We compare the performance of MDL, best-fit-I, and best-fit-II filtered by CMI and MDL both on simulated networks and on an artificial model of budding yeast. The results show that, as determined by the distance metrics $\mu_{\mathrm{ham}}^{e}$ and μ^ssd^, MDL-based filtering does not degrade inference performance, can improve inference performance, and is more effective than CMI-based filtering. Moreover, the combination of MDL filtering with the best-fit algorithm can even outperform the MDL algorithm alone. Additionally, applying MDL-based filtering is computationally less burdensome than using the MDL algorithm alone because calculating the data-coding length *L*_*D*_ is more complex than calculating the error estimate of the best-fit algorithm, and the complexity of the calculation increases dramatically as the sample size *m* increases. Last but not the least, MDL-based filtering can also be applied to the results of other minimal error algorithms such as CoD.

## Authors’ original submitted files for images

Below are the links to the authors’ original submitted files for images. Authors’ original file for figure 1 Authors’ original file for figure 2
