# Supplementary figures and images for: Using the minimum description length principle to reduce the rate of false positives of best-fit algorithms
Source: EURASIP J Bioinform Syst Biol. 2014 Jul 3;2014:13. doi: 10.1186/s13637-014-0013-2 (PMC5270450; doi:10.1186/s13637-014-0013-2)

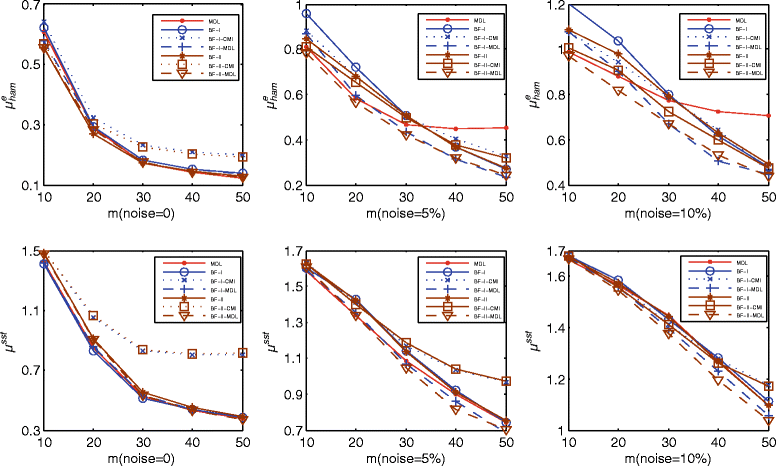

Supplement: Supplementary file 1 — Authors’ original file for figure 1 [file 13637_2014_13_MOESM1_ESM.gif]

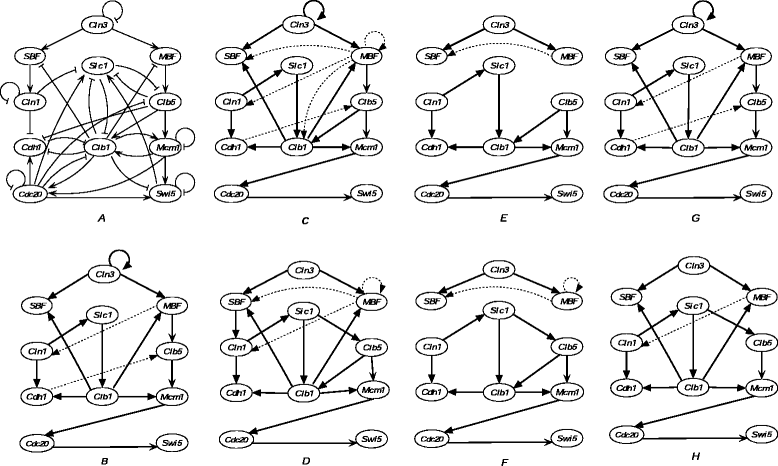

Supplement: Supplementary file 2 — Authors’ original file for figure 2 [file 13637_2014_13_MOESM2_ESM.gif]
